# New developments in AMPK and mTORC1 cross-talk

**DOI:** 10.1042/EBC20240007

**Published:** 2024-11-18

**Authors:** William J. Smiles, Ashley J. Ovens, Bruce E. Kemp, Sandra Galic, Janni Petersen, Jonathan S. Oakhill

**Affiliations:** 1 Metabolic Signalling Laboratory, St. Vincent’s Institute of Medical Research, Fitzroy, VIC 3065, Australia; 2 Research Program for Receptor Biochemistry and Tumour Metabolism, Department of Paediatrics, University Hospital of the Paracelsus Medical University, Salzburg, Austria; 3 Protein Engineering in Immunity and Metabolism, St. Vincent’s Institute of Medical Research, Fitzroy, VIC 3065, Australia; 4 Protein Chemistry and Metabolism, St. Vincent’s Institute of Medical Research, Fitzroy, VIC 3065, Australia; 5 Department of Medicine, University of Melbourne, Parkville, VIC 3010, Australia; 6 Mary Mackillop Institute for Health Research, Australian Catholic University, Fitzroy, Vic 3065, Vic. Australia; 7 Metabolic Physiology, St. Vincent’s Institute of Medical Research, Fitzroy, VIC 3065, Australia; 8 Flinders Health and Medical Research Institute, Flinders Centre for Innovation in Cancer, Flinders University, Adelaide, SA 5042, Australia; 9 Nutrition and Metabolism, South Australia Health and Medical Research Institute, Adelaide, SA 5000, Australia

**Keywords:** AMPK, cancer, mechanistic target of rapamycin, metabolism, signalling

## Abstract

Metabolic homeostasis and the ability to link energy supply to demand are essential requirements for all living cells to grow and proliferate. Key to metabolic homeostasis in all eukaryotes are AMPK and mTORC1, two kinases that sense nutrient levels and function as counteracting regulators of catabolism (AMPK) and anabolism (mTORC1) to control cell survival, growth and proliferation. Discoveries beginning in the early 2000s revealed that AMPK and mTORC1 communicate, or cross-talk, through direct and indirect phosphorylation events to regulate the activities of each other and their shared protein substrate ULK1, the master initiator of autophagy, thereby allowing cellular metabolism to rapidly adapt to energy and nutritional state. More recent reports describe divergent mechanisms of AMPK/mTORC1 cross-talk and the elaborate means by which AMPK and mTORC1 are activated at the lysosome. Here, we provide a comprehensive overview of current understanding in this exciting area and comment on new evidence showing mTORC1 feedback extends to the level of the AMPK isoform, which is particularly pertinent for some cancers where specific AMPK isoforms are implicated in disease pathogenesis.

## Introduction

For cells to maintain a stable internal state they must link growth and proliferation to availability and uptake of nutrients such as glucose, lipids and amino acids [1]. This is achieved through metabolic homeostasis, the exquisite coordination of various biochemical reactions that ensure sufficient energy supply for ATP-requiring, anabolic processes such as the synthesis of proteins, lipids and cholesterol. Cells must detect periods of nutrient or energy stress (e.g., fasting and muscle contraction) and rapidly reprogram their metabolism to ATP-generating, catabolic processes such as lipid oxidation, glycolysis and autophagy, or risk impaired cellular function and destructive oxidative stress. Dysregulation of metabolic homeostasis underpins many major and socially burdensome human diseases including cancer, Type 2 diabetes, fatty liver disease and obesity [2].

**Figure 1:**
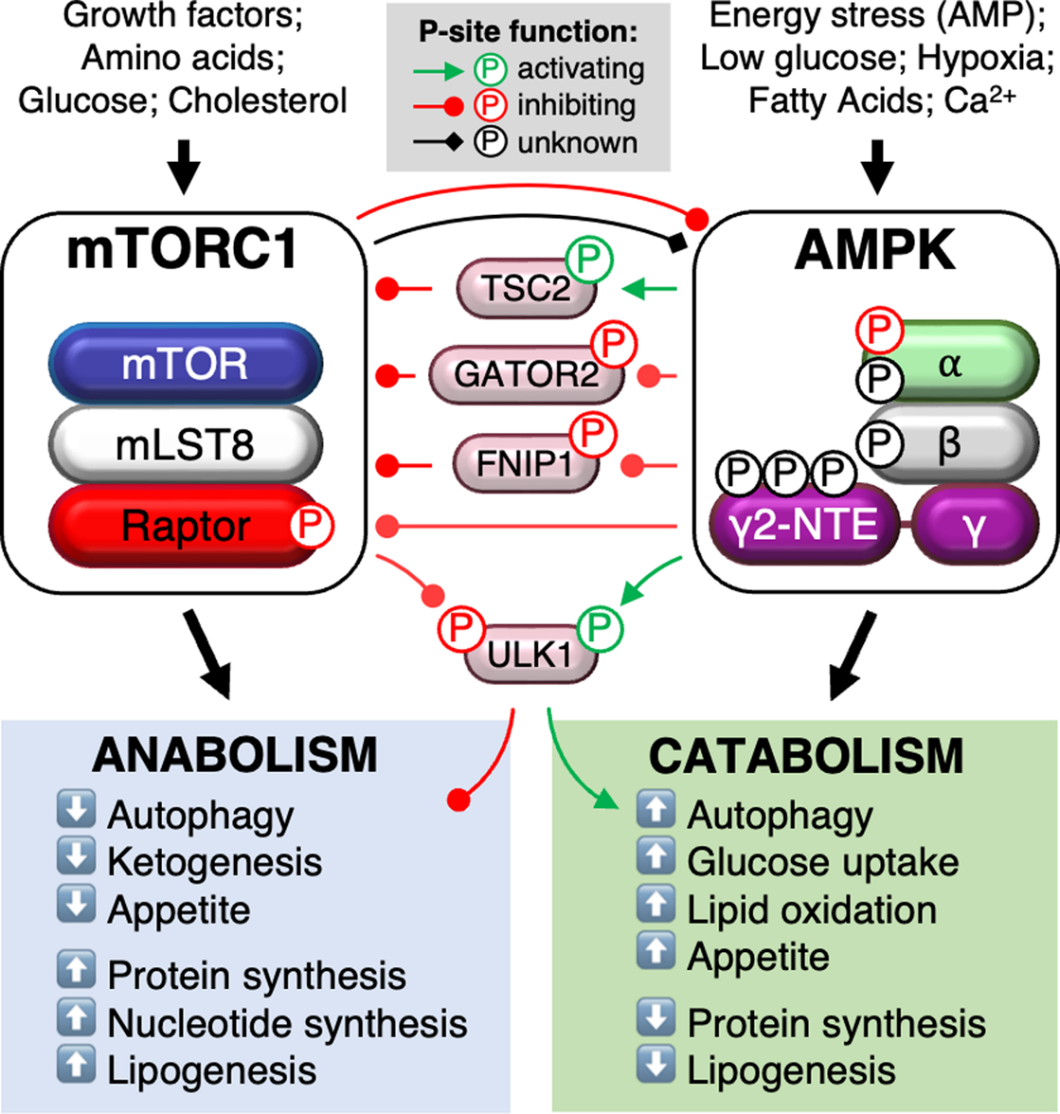
Reported pathways of AMPK/mTORC1 cross-talk In response to energy and nutrient stress, AMPK inhibits mTORC1 signalling through direct and indirect phosphorylation events. mTORC1 reciprocally supresses AMPK activity through direct phosphorylation of the α subunit (α2-S345, possibly α1-S347). Several other phospho-sites on AMPK α and β isoforms (α2-S377, β1-S182, β2-S184) may lie directly or indirectly downstream of mTORC1. The AMPK γ2 N-terminal extension (NTE) may be a hot spot for mTORC1 signalling. Classically, AMPK and mTORC1 reciprocally regulate ULK1 to modulate autophagic flux.

At the centre on nutrient-sensing and metabolic regulation are two protein kinases, AMP-activated protein kinase (AMPK) and mammalian (or mechanistic) target of rapamycin (mTOR) (Figure 1). AMPK is an αβγ heterotrimer comprised of a catalytic α-subunit and regulatory β- and γ-subunits. Multiple isoforms of each subunit exist (α1/2, β1/2, γ1/2/3), allowing for assembly of up to 12 different complexes with distinct biochemical properties and tissue expression patterns. AMPK is canonically activated by a tripartite mechanism involving AMP and ADP displacing ATP bound to the γ-subunit. First, AMP and ADP promote activating phosphorylation of a threonine residue at position 172 in the α-subunit kinase activation loop (α-pT172) and second, protect α-pT172 from dephosphorylation by phosphatases, resulting in net increase in α-T172 phosphorylation and AMPK activity. Third, AMPK already phosphorylated on α-T172 can be further allosterically activated by AMP and only modestly by ADP [3–5]. LKB1 (liver kinase B1) is considered the major α-T172 kinase [6,7], but α-T172 can be effectively phosphorylated by CaMKK2 (Ca^2+^-calmodulin-dependent protein kinase kinase 2) in response to intracellular Ca^2+^ oscillations [8]. AMPK is also activated by a range of small synthetic molecules, natural products and fatty acyl-CoAs that occupy a hydrophobic cleft termed the allosteric drug and metabolite (ADaM) site [9–13], formed between a carbohydrate-binding module in the β-subunit and the α-subunit kinase domain. The ADaM site is stabilised by phosphorylation of the β1 residue S108, a *cis*-autophosphorylation site as well as a site for the autophagy initiator unc-51-like kinase (ULK1) [9,14]. Intriguingly, purified AMPK lacking phosphorylation of α-T172 (the most abundant form of AMPK in resting cells [3,9,15]) can be activated considerably by ADaM site drugs provided β1-S108 is phosphorylated. Although AMP or ADaM site drugs alone negligibly activate AMPK complexes lacking α-pT172 *and* β1-pS108, appreciable activation of fully dephosphorylated AMPK has been achieved by simultaneous incubation with an ADaM site drug and AMP (termed synergistic activation) [9,14].

mTOR exists in at least two functionally dissimilar complexes, mTORC1 and mTORC2, each defined by unique accessory proteins, substrate phosphorylation profiles and differential sensitivity to the inhibitor drug rapamycin. The rapamycin-sensitive mTORC1 contains the scaffold protein Raptor that dictates substrate recruitment and subcellular localisation [16,17]. mTORC2 contains the unrelated scaffold protein Rictor that binds the mammalian SAPK-interacting protein 1 (mSIN1). Both complexes are nucleated by mammalian lethal with SEC13 protein 8 (mLST8) that stabilises mTORC2 but has a poorly defined function in mTORC1 [18,19]. The most intensively studied area of mTORC1 regulation is its activation at the lysosome by growth factors and nutrients, in particular amino acids. The presence of extracellular growth factors is transmitted intracellularly to mTORC1 via the tuberous sclerosis complex (TSC) made up of TSC1, TSC2 and TBC1D7. TSC2 is a GTPase-activating protein (GAP) that converts the small GTPase Rheb, an allosteric activator of mTORC1, into its inactive form thereby suppressing mTORC1 activity [20]. Phosphorylation of TSC2 by Akt releases the TSC from the lysosome, causing TSC2 to dissociate from Rheb and allowing Rheb to accumulate in its GTP-bound and active form [21,22]. Provided surplus nutrients are available, mTORC1 is recruited to the lysosomal surface by the Rag GTPases where it encounters and is allosterically activated by Rheb.

AMPK and mTORC1 exist in a negative feedback loop [23], fine-tuning each other’s activity to carefully balance catabolic (mTORC1 *inhibits*, AMPK *activates*) and anabolic (mTORC1 *activates*, AMPK *inhibits*) cellular processes such as protein turnover (synthesis and degradation) and glucose and lipid metabolism. To illustrate how critical the balance between anti- and pro-growth pathways is, cells harbouring genetic defects limiting downregulation of mTORC1 by AMPK succumb to apoptosis when subjected to energy stress [24,25]. What has also become apparent is that divergent signals transmitted by individual nutrients not only converge on the lysosome to activate AMPK and mTORC1, but in some instances the two kinases share identical regulatory binding partners. In this review, we provide an update on the current landscape of AMPK and mTORC1 cross-talk and consider what the future holds for this fundamental area of metabolic research.

## Control of autophagy and cell fate by AMPK and mTORC1 phosphorylation of ULK1

ULK1 is fundamentally responsible for kick-starting autophagy, the process by which cells form vesicular autophagosomes that sequester intracellular ‘waste’ material (e.g., long-lived proteins) for catabolism in the lysosome. AMPK and mTORC1 directly phosphorylate ULK1 at distinct sites to classically activate and inhibit autophagy, respectively [26,27] (Figure 1). Amino acid starvation is a potent inducer of autophagy, and loss of ULK1 suppression by mTORC1 rapidly drives autophagosome formation and recycling of proteins to amino acids to restimulate mTORC1 and protein synthesis [28]. However, like protein synthesis, autophagy is an energy-consuming process [29], and several reports have questioned the role of AMPK in this context [30,31]. In fact, more recent studies strongly indicate AMPK inhibits ULK1-mediated autophagy when cells are initially confronted with an energy shortage, such as removal of a major carbon source like glucose, or pharmacological activation of AMPK during amino acid starvation [29,32,33]. In this situation, AMPK was shown to protect the ULK1-associated autophagic machinery from caspase-mediated degradation, allowing autophagy to commence with energy re-balance [34]. This probably explains why AMPK dictates whether cells engage the autophagic process or succumb to apoptosis when energy stress is prolonged [31,35,36].

ULK1 phosphorylates AMPK on several sites including β1-S108, the net effect of which is reduced α-pT172 and ADaM site stabilisation, leading to the untested hypothesis that ULK1 mediates a ‘ligand switch’ from AMP to an ADaM site metabolite such as palmitoyl-CoA [14,37]. ULK1 similarly inhibits mTORC1 via direct phosphorylation of Raptor to limit substrate binding [38]. ULK1 therefore represents an extra, substrate-mediated layer of complexity to AMPK/mTORC1 cross-talk [39].

## AMPK and mTORC1 share identical lysosomal activation platforms

**Figure 2:**
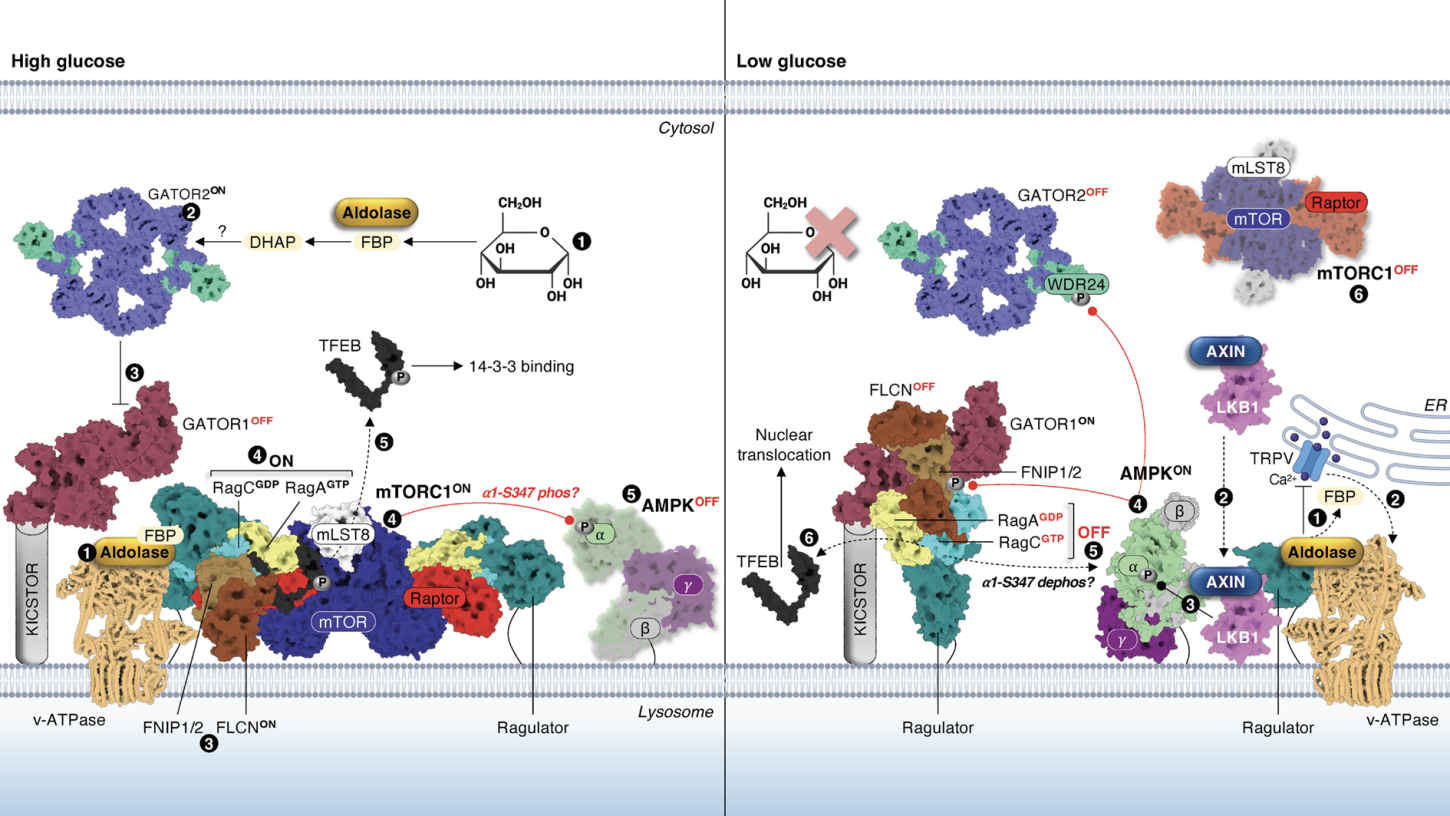
Lysosomal glucose-sensing by AMPK and mTORC1 Left panel: (1) in conditions of high glucose, aldolase associates with v-ATPase (PDB: 5VOX) and is occupied by its substrate FBP. Conversion of FBP to DHAP by aldolase results in downstream mTORC1 activation. (2) DHAP availability is conveyed to GATOR2 (PDB: 7UHY) by an incompletely defined glucose-sensing mechanism. (3) Active GATOR2 supresses the GAP function of GATOR1 (PDB: 6CES), itself anchored to the lysosome by the KICSTOR complex, resulting in loading of RagA with GTP. RagC is concurrently loaded with GDP by FLCN in a complex with FNIP1 or FNIP2. (4) Rag dimers are tethered to the lysosome by the Ragulator complex (bound to v-ATPase), and in the ‘ON’ configuration they recruit mTORC1 to the lysosomal surface via binding to the ‘claw’ apparatus of Raptor. (5) This mechanism is sufficient for mTORC1 to phosphorylate TFEB independent of growth factors, in turn resulting in TFEB 14-3-3 binding and cytosolic sequestration. Presented is the mTORC1-TFEB-Rag-Ragulator megacomplex (PDB: 7UXH) aligned with the active FLCN/FNIP2 complex (PDB: 8DHB). Activated mTORC1 potentially phosphorylates α1-S347 on a pool of AMPK (PDB: 7JHG; inactive α1β2γ1) that is a resident of the lysosome. Right panel: (1) when glucose levels fall, aldolase is vacated by FBP and inhibits TRPV at lysosome-endoplasmic reticulum (ER) contact points, attenuating local Ca^2+^ concentrations. (2) This results in TRPV associating with v-ATPase and forming a ternary complex with aldolase, in turn promoting lysosomal translocation of the AXIN/LKB1 dimer. (3) LKB1 (PDB: 2WTK) then encounters and phosphorylates α-T172 on AMPK (PDB: 4RER; active α1β2γ1). (4) Activated AMPK phosphorylates and inhibits both GATOR2 and FNIP1 (shown is the inactive FLCN-FNIP2 lysosomal complex (PDB: 6NZD) aligned with GATOR1-Rag), (5) triggering the ‘OFF’ state of the Rags by GDP loading of RagA and GTP loading of RagC, respectively. (6) mTORC1 is now inhibited, which relieves TFEB phosphorylation and induces its nuclear translocation.

The Ragulator and vacuolar H^+^-ATPase (v-ATPase) are two resident lysosomal complexes that cooperatively integrate disparate nutrient cues to direct AMPK/mTORC1 signalling (Figure 2). The Ragulator complex is a heteropentamer consisting of p18, p14, MP1, C7orf59 and HBXIP (or LAMTOR1-5), whereby palmitoylated and myristoylated LAMTOR1 anchors the entire complex to the lysosome [40,41]. The Ragulator complex tethers Rag GTPases to the lysosome, and when nutrients are in abundance this signal is relayed to the lysosomal Rags to induce formation of active heterodimers of GTP-loaded RagA or RagB bound to GDP-loaded RagC or RagD [40,42]. In this configuration, the Rags are literally grabbed by Raptor by an internal ‘claw’ that pulls mTORC1 from the cytosol to the lysosomal surface [43]. v-ATPase shuttles protons from the cytosol into the lysosomal lumen to maintain its acidity, which is critical for the function of lysosomal enzymes. It also serves as a sensor of amino acids in the lysosomal lumen (likely liberated by autophagic proteolysis) and transmits this nutritional state to the Rags and mTORC1 through a physical interaction with the Ragulator complex [42,44].

A series of studies have now uncovered non-canonical, AMP-independent mechanisms of AMPK activation by glucose starvation and low-dose metformin, which involves formation of an AXIN/LKB1 complex that translocates to the lysosome where it interacts with v-ATPase and the Ragulator complex [45–49]. This essentially colocalises LKB1 with a population of AMPK already stationed at the lysosome. The significance of AMPK and mTORC1 utilising identical lysosomal protein complexes to yield opposite metabolic outcomes is perhaps best exemplified by the effect of glucose availability, as discussed in the following section.

## AMPK inhibition of mTORC1

### The metabolic checkpoint

The first evidence of AMPK controlling mTORC1 activity was reported over 20 years ago when AMPK was found to trigger activation of TSC2 by direct phosphorylation on T1271 and S1387 (human isoform 1) [24]. The mechanism remains almost completely unknown. One possibility is that AMPK potentiates, albeit indirectly, the GAP activity of TSC2 towards Rheb, converting Rheb to its inactive, GDP-bound form that causes reductions in cell size and enhanced cell survival in the face of glucose deprivation [24]. Mutation of these AMPK sites on TSC2 to non-phosphorylatable alanine residues offset, in part, the attenuation of mTORC1 substrate p70 ribosomal S6 kinase 1 (S6K1) and eIF4E-binding protein 1 (4E-BP1) phosphorylation after AMPK activation by 2-deoxyglucose (2-DG) treatment, an inhibitor of glycolysis [24]. Potentiation of the AMPK/TSC2 module is LKB1-dependent [50,51], and it is particularly noteworthy that gastrointestinal polyps taken from *LKB1*-deficient mice display elevated mTORC1 signalling [50], providing compelling early evidence for the therapeutic potential of targeting AMPK in certain malignancies.

**Figure 3:**
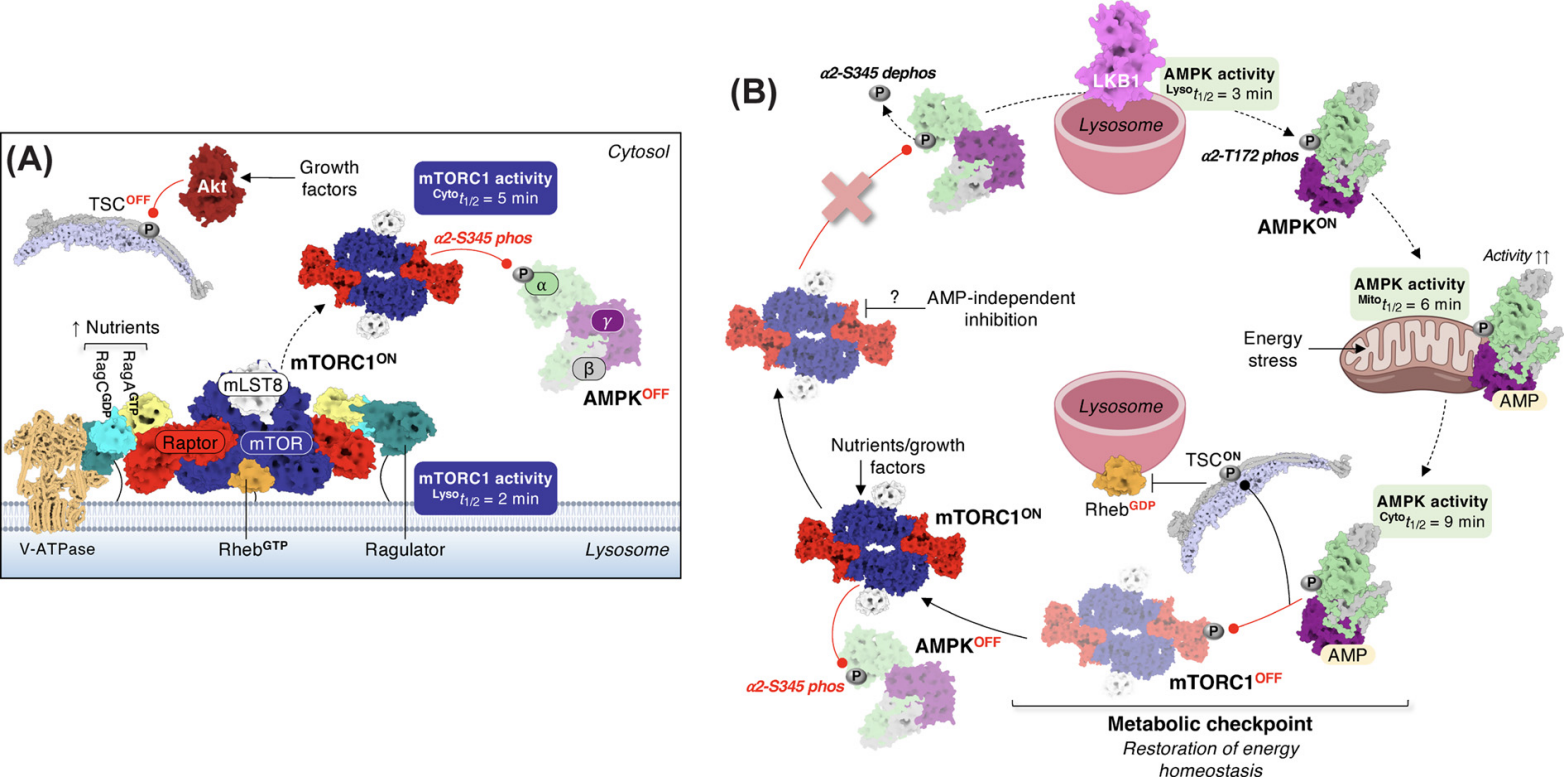
The metabolic checkpoint (**A**) Nutrient and growth factor abundance activates mTORC1 at the lysosome in a Rag- and Rheb-dependent manner, respectively. Depicted is GTP-bound Rheb and mTORC1 (PDB: 6BCU) superimposed with the Raptor-Rag-Ragulator complex (PDB: 6U62). GTP loading of Rheb requires Akt (PDB: 1O6K) phosphorylation of TSC2 and suppression of its GAP function (shown is the human TSC complex; PDB: 7DL2). The time to half-maximal lysosomal mTORC1 activity (^Lyso^*t*_1/2_) is approximately 2 min, at which point mTORC1 dissociates from the lysosomal surface and enters the cytosol where it subsequently phosphorylates α2-S345 of AMPK (^Cyto^*t*_1/2_ = 5 min), retaining the complex in its cytosolically inactive form. (**B**) Complete or partial inhibition of mTORC1, independent of overt changes to cellular AMP concentrations, causes α2-S345 dephosphorylation and lysosomal targeting of AMPK where it is activated by LKB1. This constitutes the initial step in the α2-S345-dependent AMPK signalling cascade. AMPK reaches half-maximal activity at the lysosome within 3 min [52], in which its activation kinetics are highly reminiscent of nutrient-stimulated mTORC1 at the lysosome [53]. Following its phosphorylation by LKB1, AMPK is transported to mitochondria where in the face of energy stress, AMP binding to the γ-subunit further activates the complex. Maximally activated AMPK then reaches the cytosol and completely switches off mTORC1 directly by Raptor phosphorylation and/or indirectly by TSC2 phosphorylation. This step represents the metabolic checkpoint and causes cells to discontinue cycling until the energy shortfall is corrected. Restoration of energy balance reactivates mTORC1 and AMPK is once again inhibited by α2-S345 phosphorylation until the cycle is reinitiated.

It was later observed that mTORC1 was still inhibited by AICAR and the mitochondrial complex I inhibitor phenformin in *TSC2*-null MEF cells, leading to the hypothesis that AMPK utilises alternate mechanisms to combat mTORC1 signal transduction during metabolic duress [25]. This indeed was the impetus for the second major discovery of AMPK inhibiting mTORC1 via direct phosphorylation of Raptor on S722 and S792, which triggers 14-3-3 binding and a reduction in mTORC1 kinase activity [25]. AMPK-mediated phosphorylation of Raptor signifies a metabolic checkpoint (Figure 3), whereby cells arrest in the G_1_- and S-phases of the cell cycle to ameliorate energy shortfalls before the onset of cell division [25]. Abolishing AMPK-mediated phosphorylation of Raptor elicits some degree of apoptosis following energy stress, but this is markedly exacerbated when accompanied by loss of TSC2 expression [25].

### Glucose sensing

Glucose sensing upstream of AMPK is proposed to centre around the glycolytic enzyme aldolase. The present model is that in glucose-replete conditions, aldolase occupied by its substrate fructose-1,6-bisphosphate (FBP) associates with v-ATPase to maintain its activity [45]. When glucose levels fall, aldolase is inevitably vacated by FBP and inhibits the endoplasmic reticulum transient receptor potential vanilloid (TRPV) Ca^2+^-release channel at lysosomal contact points (Figure 2, right panel) [46]. A local reduction in Ca^2+^ then allows TRPV to associate with v-ATPase where it forms a ternary complex with aldolase. This triggers a conformational change in v-ATPase that leads to the formation of AXIN/LKB1, which translocates to the lysosome, binds the Ragulator complex, and activates AMPK [45,46,49]. AXIN itself is argued to inhibit mTORC1 by disabling its interaction with the Rags [54]. By contrast, the glycolytic metabolite dihydroxyacetone phosphate (DHAP), generated from FBP by aldolase, has been flagged as a critical element recognised by the glucose-sensing machinery upstream of mTORC1 [55]. DHAP availability is transduced to mTORC1 via the GATOR2-GATOR1 apparatus that is responsible for GTP turnover of RagA/B. GATOR1 is recruited to the lysosome by another protein complex called KICSTOR and inhibits mTORC1 through its GAP activity towards RagA/B [56,57]. GATOR2, an inhibitor of GATOR1, is a pentamer whose function has largely been studied in the context of sensing of the amino acids leucine and arginine by Sestrin2 and CASTOR1, respectively [58,59]. When these amino acids are plentiful, they sequester Sestrin2 and CASTOR1 away from GATOR2, relieving inhibition of the complex, so it can activate mTORC1.

Notwithstanding some uncertainty over its relevance [55], aldolase has been deemed the sensor responsible for glucose-stimulated mTORC1 activity [60]. In that study, an aldolase mutant (D34S) which competently binds FBP but prevents enzymatic cleavage into DHAP, preserved lysosomal localisation and activation of mTORC1 in low-glucose conditions. The FBP binding-deficient aldolase mutant (K230A) blocked lysosomal mTORC1 association despite ample glucose availability [60]. These findings supported earlier reports of mTORC1 being responsive to glucose levels independently of AMPK [55,61,62]. Whilst this ostensibly pins aldolase as the universal glucose sensor for both AMPK *and* mTORC1, it does not exclude the possibility of other regulatory metabolites and/or pathway sensors responsive to differential patterns of cellular glucose utilisation [63–65]. It is also unclear whether preventing DHAP generation by aldolase (D34S mutant) simply results in DHAP alternatively being generated from glycerol. In that regard, precisely how mTORC1 is dislodged from the lysosome when aldolase is unoccupied by FBP remains unknown.

Bidirectionality of glucose sensing was confirmed by a recent investigation showing that glucose deprivation triggers AMPK-mediated phosphorylation of S155 on WDR24, a core component of the GATOR2 complex (Figure 2) [66]. AMPK-mediated phosphorylation of WDR24 disrupts GATOR2 integrity, causing lysosomal dissociation and inactivation of mTORC1 in HEK293 cells after 1 h of glucose deprivation [66]. HEK293 cells rely heavily on glycolysis for ATP production and acute glucose limitation is enough to rapidly elevate cellular AMP:ATP ratios [45]. This makes it difficult to discern whether antagonism of mTORC1 by AMPK-mediated phosphorylation of GATOR2 manifests downstream of aldolase and is contingent upon AXIN/LKB1 translocation to the lysosome. Loss of GATOR1 function has been shown to augment glucose starvation-induced phosphorylation of Raptor by AMPK, but not the classical AMPK substrate acetyl-CoA carboxylase (ACC), despite hyperactivity of mTORC1 [55]. Collectively this indicates (1) GATOR1 potentially has a role in controlling AMPK and mTORC1 colocalisation, conceivably at the lysosomal surface, and (2) Raptor phosphorylation by AMPK is insufficient to completely abolish mTORC1 kinase activity; the latter being supported by a disconnect between residual Raptor-S792 phosphorylation and recovery of mTORC1 signalling during energetic stress [67]. Nevertheless, glucose-induced mTORC1 activation was reported to also occur by O-GlcNAcylation of Raptor at T700, favouring the Raptor-Rag interaction and lysosomal localisation of mTORC1, which could be overturned by AMPK-mediated phosphorylation of Raptor [68]; similarly, AMPK phosphorylates the acetyltransferase p300 in response to nutrient limitation (including glucose withdrawal), restricting Raptor acetylation and mTORC1 activation at the lysosome [69,70]. Because a substantial portion of freshly generated ATP in glycolytic cells is partitioned to energetically costly processes like protein synthesis [71], glucose sensing and phosphorylation of GATOR2 by AMPK is likely coupled to blockade of the protein synthetic machinery even when amino acids are present.

### Transcriptional control of mitochondrial and lysosomal biogenesis

The MiT/TFE transcription factors TFEB and TFE3 are master controllers of autophagic and lysosomal gene expression as well as transcriptional activation of mitochondrial biogenesis [72]. TFEB is the better characterised and phosphorylated by mTORC1 at several sites to prevent its nuclear translocation [73–76]. Folliculin (FLCN) is a RagC-specific GAP that complexes with FLCN-interacting protein 1 (FNIP1) or FNIP2 and seems required for mTORC1-mediated phosphorylation of TFEB/TFE3 but not other substrates like S6K1 and 4E-BP1 [77–80]. mTORC1 remains bound to the lysosome even if Rheb is deactivated [81], which may explain why FLCN-directed phosphorylation of TFEB by mTORC1 occurs outside of the growth factor axis and is sensitive to prevailing nutrient levels [77]. This also reconciles FLCN's paradoxical role as a tumour suppressor, specifically by limiting the extent of TFEB-driven oncogenic transcriptional programs [77,82].

FNIP1 is phosphorylated by AMPK on up to five serine residues although not all sites are predicted to be functional [83]. Energetic stress, caused by mitochondrial poisons (e.g., the depolarising agent CCCP) and glucose deprivation, stimulates AMPK-mediated phosphorylation of FNIP1, causing inhibition of FLCN, accumulation of GTP-bound RagC and lysosomal detachment of mTORC1 (Figure 2) [83]. This releases inhibitory phosphorylation of TFEB by mTORC1, allowing it to orchestrate mitochondrial and lysosomal biogenesis [83]. Activation of AMPK by the ADaM site drug 991 in HEK293T cells expressing a non-phosphorylatable FNIP1 mutant (alanine substitutions at AMPK sites) blunted mTORC1-mediated phosphorylation of S6K1 and 4E-BP1, but not TFEB [83]. As such, a picture emerges of AMPK leveraging specific substrates to disrupt disparate branches of mTORC1 signalling. Before the AMPK-mediated restraints on cell growth and division are lifted (phospho-Raptor/TSC2), targeting of FNIP1 would ensure restoration of homeostasis by bolstering cellular bioenergetics (mitochondrial biogenesis) and disposing of constituents that threaten cell viability (lysosomal biogenesis, autophagy).

## mTORC1 inhibition of AMPK

### A novel lysosomal α2-AMPK activation pathway

A 2015 report from the Petersen lab showing nitrogen stress elicits TORC1 inhibition and activation loop (T189) phosphorylation of the AMPKα fission yeast homolog Ssp2, independent of overt changes to adenine nucleotides [84], was the catalyst for the hypothesis that mTORC1 exerts direct control over AMPK. Dephosphorylation of the conserved Ssp2 residue S367, corresponding to human AMPK α1-S347 (also termed S356 depending on the α1-AMPK variant used) and α2-S345, coincided with activation loop phosphorylation and reduced cell size at division [23]. Conversely, genetically mimicking S367 phosphorylation (S367D mutant) desensitised Ssp2 to nutrient stress and enhanced cell growth [23]. Inhibition of TORC1 in fission yeast reduced phosphorylation of Ssp2-S367, while mTORC1 directly phosphorylated mammalian AMPK on α1-S347 and α2-S345 *in vitro*; dephosphorylation of these sites augments α-T172 phosphorylation and AMPK signalling, delaying mammalian cell proliferation under nutrient stress [23,85]. Altogether these findings completed an ancient negative feedback loop between AMPK and mTORC1 to coordinate nutrient-sensing with cell growth and division (Figure 3).

The mechanism by which inhibition of mTORC1 activates AMPK involves lysosomal targeting of the heterotrimer and engagement with LKB1 [85]; however, this effect was observed in α2- and not α1-AMPK complexes [85], aligning with previous studies demonstrating α2-AMPK is the preferred substrate of LKB1 [86–89]. α2-pS345 was not affected by AMPK β-subunit myristoylation (a modification that targets AMPK to organelles like lysosomes) [23], indicating mTORC1 phosphorylates α2-S345 in the cytosol following its own dissociation from the lysosomal surface [53]. Because α2-pS345 is sensitive to growth factor abundance, this points to Rheb as the mTORC1-activating agent controlling AMPK in this scenario [23]. α2-S345-dependent lysosomal targeting is also extremely transient (<1 min) [85], suggesting that following α-T172 phosphorylation, AMPK is rapidly redistributed to subcellular compartments populated by certain classes of substrates. In support of that assertion, α2-S345 dephosphorylation alone, in the absence of energy stress, strongly promotes AMPK-mediated phosphorylation of ACC, and to a lesser extent ULK1, but has weak effects toward TSC2 and Raptor [23].

α2-S345 is situated in the α-linker region containing essential AMP-sensing modules termed α-RIM1 and α-RIM2 [90]. α2-S345 also borders a so-called R365 pocket implicated in AMP-mediated allosteric AMPK activation and protection of α-pT172 against dephosphorylation [91–93]. Proximity of α2-S345 to major AMP-sensing regulatory elements in AMPK might explain, in part, why dephosphorylation of this site only restricts cell proliferation when either nutrients are limiting (e.g., amino acids) or energy stress is provoked (e.g., 2-DG) [23,85]. One possibility, although this remains to be tested, is that unlike current dogma that states AMP *promotes* α-pT172, mTORC1 inhibition and α2-AMPK lysosomal targeting may precede AMP occupancy of the γ-subunit. For instance, in response to metabolic stress, AMPK is activated at the lysosome more rapidly than at mitochondria and in the cytoplasm, reminiscent of the kinetics of mTORC1 activation (indicated in Figure 3) [53]; herein, the kinetics of lysosomal AMPK activity are LKB1-dependent [52]. If this novel lysosomal pathway ultimately commissions α2-AMPK to accumulate at bioenergetic hubs like mitochondria, then the complex would be perfectly poised to become maximally activated by AMP and shut down cell growth and proliferation. Whether the latter is through reinforced inhibition of mTORC1 (i.e., phospho-TSC2/Raptor) or other clusters of α2-AMPK-specific substrates, remains unresolved. Other questions relate to the initial cues responsible for disabling mTORC1-mediated sequestration of α2-AMPK to the cytosol, and the degree to which they are AMPK-dependent (e.g., glucose deprivation limiting DHAP).

### A putative lysosomal α1-AMPK activation pathway

**Figure 4:**
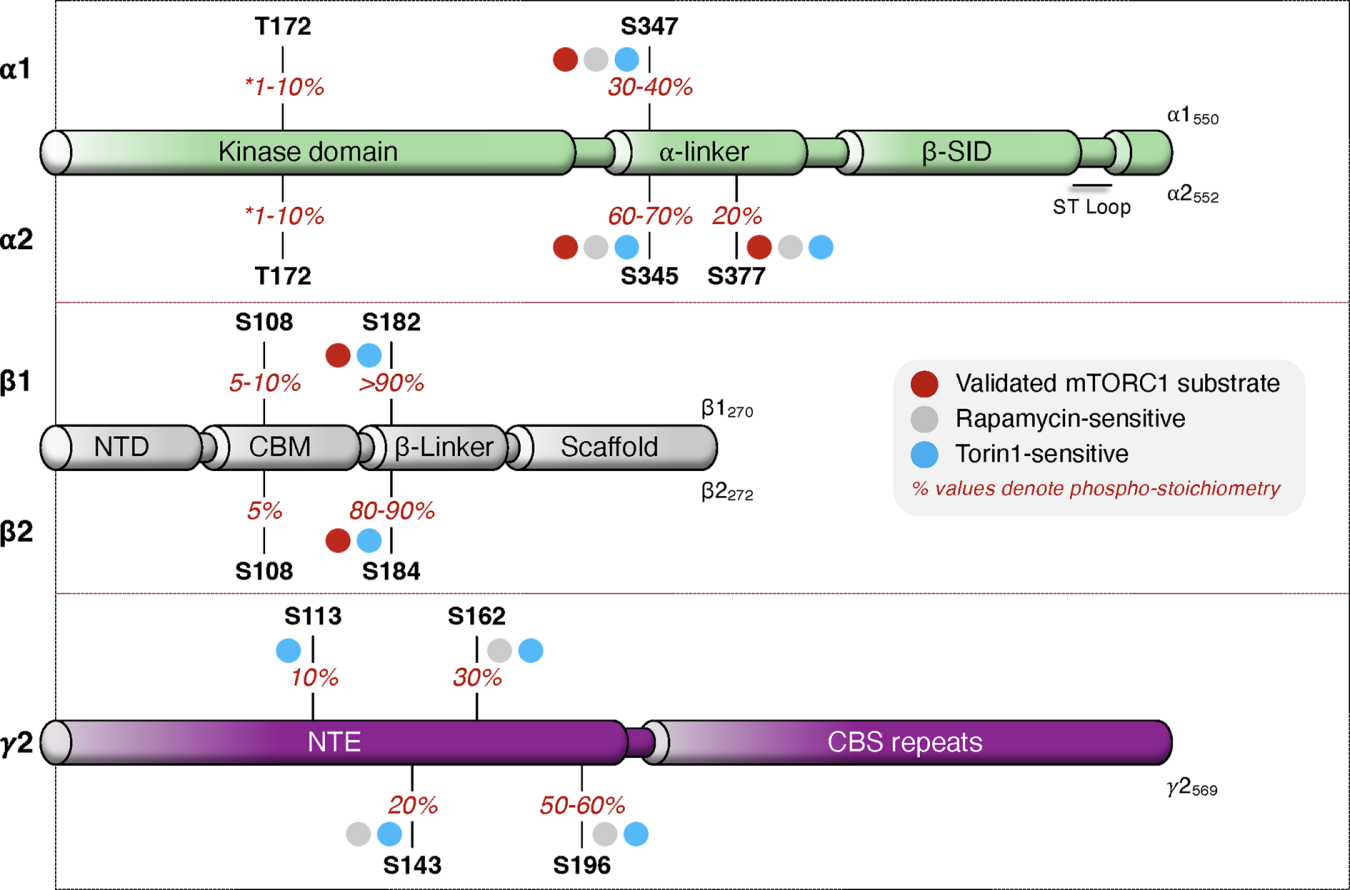
Novel torin1-sensitive phosphorylation sites on AMPK Quantitative mass spectrometry has resulted in the identification of several novel, torin1-sensitive phosphorylation sites on the β-subunit (β-S182/4) and γ2 N-terminal extension (NTE; γ2-113, γ2-S143, γ2-162, γ2-S196) of AMPK, which display variable stoichiometry levels and sensitivity to rapamycin [96]. In addition to already established mTORC1 substrates, β-S182/4 has been validated as a direct target of mTORC1 and contains the highest phospho-stoichiometry of all detected sites (>80%). Presented stoichiometry measurements were taken at baseline (i.e., pro-growth conditions; nutrient- and growth factor-rich medium) with the exception of α-T172. Asterisks indicate that baseline α-T172 phosphorylation has been measured between 1 and 10% [9,15]. Differences in α isoform phospho-stoichiometry of T172 have not been determined, although α2-T172 phosphorylation (detected by immunoblot) is reported to be ∼3-fold lower versus α1-T174 in MEF cells cultured in growth media [85]. Major structural features of each AMPK subunit are presented as a linear tube representation and for simplicity, not all subunit domains are included in the schematic.

As previously described, TFEB is phosphorylated and inhibited by mTORC1 at the lysosome in a growth factor/Rheb-independent manner [77]. This raises the question of whether a fraction of AMPK, posited to be constitutively localised to lysosomes [94], is inhibited by mTORC1. For example, we and others have shown that AMPK is a lysosomal resident even under growth conditions [49,85]. In HEK293T cells, basal stoichiometry of α2-pS345 is more than double that of α1-pS347 (∼70% vs. ∼30%; Figure 4). α1-S347 is almost completely dephosphorylated by prolonged pharmacological mTORC1 inhibition but α2-pS345 persists, indicating another kinase(s) targets this site [85]. Candidates include GSK3β [85] and CDK4 [95]; however, these await rigorous cellular validation.

In contrast to α1-S347 dephosphorylation having little impact on AMPK activity in MEFs [85], introduction of the α1-S347A mutant in the glycolytic colorectal cancer cell line HCT116 (where α1 is the predominant α isoform) *enhanced* basal AMPK activity and ACC phosphorylation [97]. The activity of this AMPK mutant is predictably increased further after 6 h of metformin exposure, but declines (compared with wild-type) after 24 h, causing an exaggerated cellular growth defect [97]. The reason(s) for mutant α1-S347A blunting AMPK activation in response to prolonged metformin exposure is unclear and may relate to intrinsic impediments in nucleotide sensing and/or an inability to reengage with the lysosome and undergo another activation cycle once α-T172 is dephosphorylated. Regardless, the impact of mTORC1-mediated phosphorylation on α-S345/7 clearly has mechanistically distinct outcomes that vary across metabolically heterogeneous cell types.

Collectively, observations with α1-AMPK (complete loss of S347 phosphorylation with torin1 and S347 dephosphorylation failing to induce lysosomal translocation) imply that α1 complexes colocalise with mTORC1 and may form a significant proportion of the lysosomal resident AMPK pool.

Whether it is at the lysosome that α1-AMPK is inhibited in a manner analogous to phosphorylation of TFEB by mTORC1 (i.e., Rheb-independent, FLCN/Rag-dependent) is unclear, although based on current evidence this is unlikely to be the case for the following reasons. Unlike TFEB, α1-S347 is a rapamycin-sensitive mTORC1 substrate [85]. As only a partial and allosteric inhibitor of mTORC1, rapamycin fails to completely block phosphorylation of all mTORC1 substrates, with TFEB being one of them [98,99]. Similar to the effect observed in cells lacking *FLCN*, TFEB is dephosphorylated and accumulates in the nucleus of *TSC2*-deficient cells in spite of Rheb and mTORC1 hyperactivity [100,101], reinforcing the notion that TFEB regulation is completely insensitive to mTORC1 activation by Rheb. Paradoxically, both mTORC1 inhibition by rapamycin and silencing of *Rheb* reverse this effect in *TSC2*-null cells, sending TFEB back to the cytoplasm and promoting its re-phosphorylation by mTORC1, for which the latter event appears to be controlled by the Rags [101]. Exactly how rapamycin and *Rheb* silencing induce TFEB nuclear export and inhibition by mTORC1 is unclear, but at the very least these findings suggest loss of Rheb switches off mTORC1 in a similar (and thus incomplete) manner to rapamycin. When considering the rapamycin-sensitivity of α1-pS347, it follows that this site is regulated by Rheb-dependent mTORC1 activation, yet without eliminating the possibility of phosphorylation and α1-AMPK inhibition at the lysosome.

### Glucose sensing

Another validated mTORC1-mediated phosphorylation site on AMPK is α2-S377, which like α-S345/7 is also highly conserved across evolution [102]. Phosphorylation of α2-S377 was first identified in two high-throughput studies [103,104], and later found to be performed by CDK1 with implications for mitotic progression [105]. Elevated α2-pS377 was also detected in human skeletal muscle during recovery (4 h) from a single bout of physical exercise [102] and further enhanced by administration of mTORC1-activating insulin, where it positively correlated with glucose uptake [102]. In that study, MEFs expressing only α2-S377A AMPK had reduced cell proliferation versus wild-type during glucose deprivation [102], replicating the effect of the α2-S345A mutant in similar conditions. A subsequent study demonstrated α2-pS377 is substantially reduced immediately after different exercise modalities, coinciding with diminution and elevation of mTORC1 and AMPK signalling, respectively [106]. α2β2γ3 AMPK is unequivocally the most exercise-sensitive AMPK complex in human skeletal muscle and γ3 complexes have the highest levels of basal α2-pS377 in mammalian cells [96,107]. Moreover, γ3-AMPK is required for insulin-independent glucose uptake and resynthesis of skeletal muscle glycogen stores in the acute recovery phase from exercise [108], which may precede mTORC1-dependent anabolism [109,110]. Thus, initial post-exercise changes in α2-pS377 may be largely confined to α2β2γ3 and followed by mTORC1 targeting the other human skeletal muscle α2-AMPK complex α2β2γ1 that has contrasting roles in glucose uptake to α2β2γ3 [91,111].

These findings imply α2-S345 and α2-S377 dephosphorylation have comparable functional effects on AMPK, especially since α2-S377 also resides near the α-linker α-RIM2. However, activities of AMPK α2-S377N (a mutation found in melanoma), isolated from HEK293 cells under basal or phenformin-treated conditions, were unchanged from wild-type. Thus, further interrogation of the function of phospho-turnover at α2-S377 under a range of metabolic conditions is warranted. In the human exercise study [102], α2-S377, α-T172 and AMPK substrates were co-phosphorylated post-exercise but became uncoupled in response to insulin. Since p-TSC2 was unchanged post-exercise, the only presented theory for α2-pS377 function alludes to a shift in AMPK substrate selectivity to allow concurrent AMPK and mTORC1 activity [102]. Precisely how mTORC1 inhibition and dephosphorylation of α2-S377 is coupled to glucose sensing and cell proliferation remains enigmatic.

### New mTORC1 sites on AMPK

Mass spectrometry has been used to quantify the stoichiometry of phosphorylation sites across the 12 AMPK heterotrimers transiently expressed in HEK293T cells [96]. This analysis identified several novel torin1-senstive phosphorylation sites on the β-subunit (β1-S182, β2-S184) and γ2-subunit isoform (γ2-S113, γ2-S143, γ2-S162, γ2-S196) displaying variable phosphorylation stoichiometries and responsiveness to rapamycin (Figure 4). These sites all contain an mTORC1-favoured proline at the P+1 position and β-S182/4 has been independently confirmed as an mTORC1 substrate, at least *in vitro* [102,96]. Furthermore, in keeping with observations in rodent skeletal muscle and liver [112,113], β-S182/4 has by far the highest phospho-stoichiometry under basal conditions, in some instances approximating 100% for β1-containing AMPK. A non-phosphorylatable mutation of this site elevated nuclear activity of β2- but not β1-AMPK, in turn potentiating cell proliferation under nutrient stress, which notably is the opposite effect of α2-S345A and α2-S377A substitutions. Each γ2 site is situated in a unique N-terminal extension (NTE) unrelated to other γ isoforms. The γ2-NTE has previously been shown to promote phosphorylation of α-T172 [114], raising the possibility these putative mTORC1 substrates modulate AMPK activity. Phosphorylation of γ2-S196 in particular, which has the highest stoichiometry of the four γ2 sites (∼50-60%), has been shown in separate high-throughput studies to be upregulated by insulin and inhibited by torin1 in adipocytes, and modulated by glucose levels in pancreatic β-cells [115,116]. The human γ2-NTE contains 14 serine-proline sites and may be a regulatory hotspot for AMPK complexes containing this isoform. Understanding the function of these phospho-sites and interplay with mTORC1 signalling has physiological significance when considering constitutive activation of γ2-AMPK is associated with a variety of disease states [117,118]. It will also be important to delineate how mTORC1 accesses these and α-subunit substrates. Canonical mTORC1 substrates contain a five residue TOR signalling (TOS) motif required for Raptor binding (e.g., S6K1: FDIDL; 4E-BP1: FEMDI; PRAS40: FVMDE) [119–121]. Candidate TOS motifs on AMPK include a conserved segment in the β-subunit (FEVFD; β1/2: 160-164) situated adjacent to the β-S182/4 phospho-sites. Pinpointing these regulatory mechanisms will shed light on the physiological conditions by which mTORC1 phosphorylates AMPK.

## Implications for disease

Dysregulation of AMPK-mTORC1 cross-talk obviously has implications for diseases and indications with metabolic dimensions but the relationship is far from clear. For example, AMPK opposes tumour progression by constraining oncogenic mTORC1 signalling but also endows tumours with a survival advantage in nutrient-poor microenvironments. Answers may lie in the specific and context-dependent roles of AMPK isoforms. α1- and β2-AMPK are both up-regulated in several cancers [122,123] and α1 contributes to the progression of cervical, pancreatic and colorectal cancers [124–126]. Alternatively, α2-AMPK may prevent metastasis in certain cancers and is frequently down-regulated in skin cancer [127,128]. Conversely, down-regulation of α1 expression and up-regulation of α2-AMPK activity both promote breast cancer metastasis [129,130]. Clearly lacking is a comprehensive exploratory analysis of how AMPK isoforms are regulated in cancer, and whether their personalised relationships with mTORC1 regulates tumour progression. For example, does preferential activation of α2-AMPK by LKB1 (itself a tumour suppressor), culminating from mTORC1 inhibition, contribute to its anti-tumourigenic standing? Equally, does AMPK contribute (i.e., by phosphorylation of FNIP1) to the pro-tumour effects of TFEB in certain malignancies? In parallel, is the pro-growth effect of β2-S184 dephosphorylation in the nucleus utilised by metabolically-stressed tumours? mTORC1 can be activated in the nucleus [131], but where β2-S184 phospho-turnover occurs in the cell is unknown. Finally, AMPK directly activates mTORC2 to promote cell survival during energetic stress [132], a mechanism that may contribute to tumourigenesis and drug resistance [133]. These findings, combined with the complexities of AMPK/mTORC1 feedback mechanisms, makes it even more pertinent to uncover AMPK isoform signalling-specificity not only in cancer, but a range of conditions where aberrant mTOR activity is implicated in disease pathogenesis.

As a case in point, α1β2-containing complexes account for the bulk of AMPK activity in human adipose tissue and contribute to adipogenesis [134], whereas genetic loss of adipose tissue mTORC1 activity in mice causes systemic hyperlipidaemia, progressive lipodystrophy and fatty liver disease [135,136]. Conversely, mTORC1 is known for its involvement in diabetes progression [137], and final considerations concern the timing of α2-AMPK and mTORC1 activity in skeletal muscle in response to exercise, and relative inputs from both kinases (e.g., kinetics of α2-S377 phosphorylation) contributing to optimal glucose handling and homeostasis and insulin sensitivity.

## Conclusion

AMPK and mTORC1 exist in an ancient negative feedback loop and serve as metabolic rheostats that couple intracellular nutrient availability and systemic factors to the growth potential of the cell. There is substantial variability in terms of how AMPK and mTORC1 regulate each other, a likely manifestation of cellular metabolic heterogeneity, subcellular localisation of the two kinases, and in the case of AMPK, different isoforms assembling in the heterotrimer. We are only just beginning to understand how their interplay has broader physiological significance in health and disease. Delineating these regulatory subtleties will be no small feat and necessitates future, collaborative efforts amongst experts across both fields.

## Summary

- Despite operating antagonistically, AMPK and mTORC1 interact with identical lysosomal complexes, whereby bidirectional glucose sensing appears to be at least one point of convergence.
- Inhibition of mTORC1 signalling networks by AMPK is executed by at least four distinct mechanisms: phosphorylation of TSC2 and Raptor to induce a growth arrest and cell survival, phosphorylation of FNIP1 to inhibit FLCN that leads to preferential activation of TFEB, and phosphorylation and destabilisation of GATOR2 when cellular glucose supply is limited.
- mTORC1 directly inhibits AMPK by phosphorylating α2-S345 to prevent lysosomal targeting and activation by LKB1. Other mTORC1 substrates on AMPK, α1-S347 and α2-S377, seemingly control AMPK activity, but the mechanisms are unresolved.
- There are a range of novel phosphorylation sites on AMPK sensitive to pharmacological mTORC1 inhibition, including β1-S182 and β2-S184, as well as several in the γ2-NTE that have unknown function.
- Unravelling the intricacies of AMPK/mTORC1 cross-talk will contribute to therapeutic advances in diseases like cancer, informing decisions over whether to take advantage of AMPK activators or inhibitors and potential efficacy alongside conventional treatment.

## Abbreviations

2-DG: 2-deoxyglucose
4E-BP1: eIF4E-binding protein 1
ACC: acetyl CoA carboxylase
ADaM: allosteric drug and metabolite
AMPK: AMP-activated protein kinase
CaMKK2: Ca^2+^-calmodulin-dependent protein kinase kinase 2
CASTOR1: cytosolic arginine sensor for mTORC1
CDK: cyclin-dependent protein kinase
DHAP: dihydroxyacetone phosphate
FBP: fructose-1,6-bisphosphate
FLCN: folliculin
FNIP: folliculin-interacting protein
GAP: GTPase-activating protein
GSK3β: glycogen synthase kinase 3β
LAMTOR: late endosomal/lysosomal adaptor and MAPK and mTOR activator
LKB1: liver kinase B1
mLST8: mammalian lethal with SEC13 protein 8
mSIN1: mammalian SAPK-interacting protein 1
mTOR: mammalian target of rapamycin
NTE: N-terminal extension
Raptor: regulatory-associated protein of mTOR
Rheb: Ras homologue enriched in brain
Rictor: rapamycin-insensitive companion of mTOR
S6K1: p70 ribosomal S6 kinase 1
TBC1D7: TBC1 domain family member 7
TFEB: transcription factor EB
TOS: TOR signalling motif
TRPV: transient receptor potential vanilloid
TSC: tuberous sclerosis complex
ULK1: Unc-51-like kinase 1
v-ATPase: vacuolar H^+^-ATPase
